# Decreased expression of stomatin predicts poor prognosis in HER2-positive breast cancer

**DOI:** 10.1186/s12885-016-2681-7

**Published:** 2016-08-30

**Authors:** Chin-Yau Chen, Chih-Yung Yang, Yen-Chung Chen, Chia-Wen Shih, Su-Shun Lo, Chi-Hung Lin

**Affiliations:** 1 Institute of Microbiology and Immunology, National Yang-Ming University, 155, Sec.2, Li-Nong St, Taipei, 11221 Taiwan, Republic of China; 2 Department of Surgery, National Yang-Ming University Hospital, Yilan County, Taiwan, Republic of China; 3 Department of Education and Research, Taipei City Hospital, Taipei, Taiwan, Republic of China; 4 Department of Pathology, National Yang-Ming University Hospital, Yilan County, Taiwan, Republic of China; 5 Department of Pathology, Lotung Poh-Ai Hospital, Yilan County, Taiwan, Republic of China

**Keywords:** Breast cancer, Stomatin, HER2, Tumor biomarkers

## Abstract

**Background:**

Human epidermal growth factor receptor-2 (HER2) is a transmembrane tyrosine kinase receptor that is overexpressed in 25 to 30 % of human breast cancers and is preferentially localized in lipid rafts. Stomatin is a membrane protein that is absent from the erythrocyte plasma membrane in patients with congenital stomatocytosis and is the major component of the lipid raft.

**Results:**

In a total of 68 clinical cases of HER2-positive breast cancer, the absence of stomatin expression was associated with a decreased 5-year survival (65 % vs. 93 %, *p =* 0.005) by survival analysis. For stage I-III HER2-positive breast cancer, the absence of stomatin expression was associated with a decreased 5-year disease-free survival (57 % vs. 81 %, *p =* 0.016) and was an independent prognostic factor by multivariate analysis. Negative stomatin expression predicts distant metastases in a hazard ratio of 4.0 (95 % confidence interval from 1.3 to 12.5).

**Conclusions:**

These results may suggest that stomatin is a new prognostic indicator for HER2-positive breast cancer.

## Background

Human epidermal growth factor receptor-2 (HER2) is an important transmembrane tyrosine kinase receptor that is overexpressed in 25 to 30 % of human breast cancers [1]. The HER2 receptor is able to promote cell proliferation and is preferentially localized in lipid rafts, which are special sphingolipid-rich and cholesterol-rich membrane microdomains; these microdomains control activation HER2 by decreasing HER2 homodimerization and lowering the subsequent spontaneous activation of the receptor [2]. Trastuzumab (Herceptin®) is a humanized monoclonal antibody that binds to HER2 and inhibits the proliferation and survival of HER2-positive breast cancers [3].

Stomatin is a membrane protein that is absent from the erythrocyte plasma membrane in patients with congenital haemolytic anaemia or stomatocytosis [4, 5]. Northern blot analysis has revealed a widespread cellular distribution of stomatin in reticulocytes, bone marrow, kidney, brain, gut and heart as well as various cell lines [6]. Stomatin is the major component of the lipid raft in the plasma membrane of epithelial cell lines, erythrocytes, and platelet alpha granules [7–11]. Two of the few well-known functions of stomatin are, firstly, the direct modulation of the activity of the acid-sensing ion channel and, secondly, the control of glucose transporter type 1 activity [12, 13]. In addition, it has been shown that hypoxia up-regulates stomatin expression in the cerebral cortex of rats and alveolar epithelial cells [14, 15]. However, since the discovery of the stomatin in 1982, the function of stomatin across a range of different tissues still remains unknown [4, 6, 16].

Stomatin has been shown to have decreased expression in cancer cells [17]. According to the Swedish Human Protein Atlas project, immunohistochemical analysis of stomatin protein expression reveals that more than 75 % of normal breast glandular and myoepithelial cells are strongly positive for this protein [18]. In contrast, in breast cancer, the expression of stomatin in these cells was 31 % (7/23) negative, 39 % weak (9/23), 26 % moderate (6/23), and 4 % (1/23) strong positive when tissue microarrays were analyzed by immunohistochemistry [18].

Although stomatin is expressed in a significant proportion of breast cancers, the relationship between stomatin expression and breast cancer has not been explored in detail. Recently reported by Arkhipova and colleagues in 2014, stomatin is down-regulated in non-small cell lung cancer and is associated with lymph node metastases [19]. This is the first and the only one study to demonstrate that stomatin has a role in carcinogenesis. In comparison, stomatin-like protein 2, which shows a high degree of sequence similarity to stomatin, had been reported to be associated with a decreased overall survival among breast cancer [20], pulmonary squamous carcinoma [21], glioma [22], endometrial adenocarcinoma [23], laryngeal squamous carcinoma [24], esophageal squamous carcinoma [25] and colorectal cancer [26] patients.

Stomatin is the major component of lipid raft where HER2 is known to be clustered and therefore it seems likely that stomatin expression may have an impact on the pathology of HER2-positive breast cancer. In the present study, the relationship of stomatin expression and the clinical survival outcome was explored for patients with HER2-positive breast cancer.

## Methods

The archival formalin-fixed paraffin-embedded tissue samples obtained from women diagnosed of infiltrating ductal carcinoma of female breast from 2001 to 2012. The women of histologies other than infiltrating ductal carcinoma were excluded. All HER2-positive cases were either HER2 immunohistochemistry 3+ or 2+ (medium positive) which was further confirmed by fluorescence in situ hybridization (FISH) to identify HER2 gene amplification [27]. There were 5 cases excluded where the HER2 immunohistochemistry results were 2 + but the FISH studies failed. Tumor grade was defined according to the (Scarff) Bloom-Richardson (BR) grading system. The results for ER, PR, and HER2 were obtained from the medical records. Cases where ER and PR were found in more than 5 % of the tumor cells were considered to be positive. Cancer staging was based on the American Joint Committee on Cancer (AJCC seventh Edition). All the patients were operated by either one of the two breast surgeons (C-Y Chen and S-S Lo). Chemotherapy was given according to the institutional guidelines and policy of National Health Insurance Administration in Taiwan. Anthracycline chemotherapy was unrestricted but taxane chemotherapy was insurance-paid only for patients whose cancer was locally advanced or metastatic. For targeted therapy, palliative trastuzumab therapy was insurance-paid when distant metastasis occurred. In patients without a distant event, adjuvant trastuzumab was insurance-paid for patients with positive lymph node status and this policy was only effective after 2010. The duration of trastuzumab therapy was allowed for 1 year at most. In this study, no patient had ever received targeted therapy other than trastuzumab. The study was held in the National Yang-Ming University Hospital, located in the north I-Lan County, Taiwan. The clinical outcomes of the patients were surveyed until December 31, 2014. Institutional review board approval was obtained before acquisition of patient health information.

Tissue sections (4 μm thick) were subjected to heat-induced antigen retrieval in the presence of 0.01 M sodium citrate buffer, pH 6.0 for 30 min using a pressure boiler. Immunohistochemical staining was performed using a polyclonal primary antibody against stomatin (Atlas, HPA011419, Uppsala, Sweden) that was diluted at 1:400; incubation took place for 30 min. Positive control was selected from breast cancer tissue of known strong positive immunoreactivity for stomatin expression. Negative control experiments were conducted by replacing the primary antibody with phosphate-buffered saline.

**Fig. 1:**
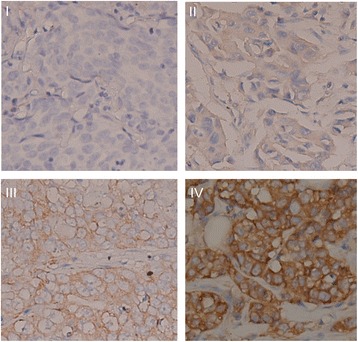
Immunohistochemical staining of breast cancer tissues. I–IV Expression of stomatin in breast cancer tissue showing the intensity score. I) Intensity score 0, no brown particles in the tumor cytoplasm or cell plasma membrane. II) Intensity score 1, faint brown staining of cell membrane. III) Intensity score 2, weak but definite brown staining of cell membrane. IV) Intensity score 3, deep brown staining of cell membrane with staining of cytoplasm

Immunostaining was scored by the researcher (C-Y Chen) and the junior pathologist (Y-C Chen), who were blinded to the patients’ outcome and other clinicopathological parameters. Discordant scores were reevaluated by the senior pathologist (C-W Shih), and a consensus score was used for further analysis. Two features, intensity and extent of immunoreactivity, were assessed as described in a previous report by Chang and colleagues [21]. The intensity of the immunostaining was classified in four categories: 0, no brown particles in the tumor cytoplasm or cell membrane; 1, faint brown staining of cell membrane; 2, weak but definite brown staining of cell membrane; and 3, deep brown staining of cell membrane together with staining of cytoplasm (Fig. 1). The percentage of positive cells were determined and classified into four groups: 1, fewer than 25 % positive tumor cells; 2, 25 to 50 % positive tumor cells; 3, 51 to 75 % positive tumor cells; and 4, more than 75 % positive tumor cells. The immunostaining index was the product of the two scores. We produced time-dependent receiver operating characteristics (ROC) curves [28, 29] for evaluation of the immunostaining indices. Area under curve (AUC) at 2- and 5-year survival was 0.765 and 0.543, respectively, suggesting a decrease in the statistical power of stomatin immunoreactivity over time, which may be explained by the early relapse of the HER2-positive breast cancer. The optimal cutoff values were 2 for 5-year-survival ROC and 7 for 2-year-survival ROC. Therefore, we defined positive expression of stomatin protein as a staining index of 4 or more, while a staining index from 0 to 3 was indicative of negative stomatin expression.

Statistical analyses were performed using STATA for Windows 10.0 (StataCorp, College Station, TX). The Student’s *t* test and Fisher’s exact test were used for statistical analysis as appropriate. We estimated the survival curves using the Kaplan-Meier product limit method [30]. Breast cancer death was defined as death related to distant metastases. Distant disease-free survival was defined as time to distant metastasis, excluding local or regional recurrence. The log-rank test was used to assess the association of survival with stomatin expression. Cox regression analysis was performed to compute hazard ratios and 95 % confidence intervals (CI) and to evaluate the effects of confounding factors during the multivariate analysis. For all statistical tests, *p* < 0.05 was considered to be significant. All *p* values were two-sided.

## Results

Using 68 HER2-positive and 58 HER2-negative samples of infiltrating ductal carcinomas of the female breast, stomatin protein expression was found to be localized mainly in the plasma membrane and partially in the cytosol. For HER2-positive patients, the overall immunohistochemistry staining results showed weak or absent staining (staining index <4) in 32 cases (47 %) and positive staining (staining index ≥4) in 36 cases (53 %). There was no statistical difference in patient age, cancer grade, cancer stage, and expression of estrogen receptor/progesterone receptor among women of positive stomatin expression compared with those of negative stomatin expression (Table 1). There was not statistical difference in types of surgery (mastectomy vs. lumpectomy) (Table 1). Most women received an anthracycline-based chemotherapy as first-line adjuvant chemotherapy. For women of HER2-positive cancers, there were 4 received CMF chemotherapy (classical CMF: cyclophosphamide, methotrexate, and fluorouracil) and one woman received taxane only because of heart disease. There were 5 women of HER2-positive cancer did not receive any adjuvant chemotherapy at all, including 2 women of early stage and 3 women who refused chemotherapy. There was not statistical difference in types of chemotherapy (Table 1). The proportions of patient who had ever received trastuzumab therapy were 42 % (15/36) in stomatin-positive group and 47 % (15/32) in stomatin-negative group, where there was no statistical difference in the proportions in receiving trastuzumab (Table 1).

**Table 1:** Patient age, tumor characteristics, hormone receptor status, and systemic treatment in relation to stomatin expression in women with HER2-positive breast cancers (*n* = 68) and HER2-negative breast cancers (*n* = 58)

Patient Characteristics	HER2-positive			HER2-negative		
	Stomatin (+) (*n* = 36)	Stomatin (−) (*n* = 32)	*p* values	Stomatin (+) (*n* = 38)	Stomatin (−) (*n* = 20)	*p* values
Mean age (year)	53	58	0.109*	54	52	0.659*
Grade						
I	6	4	0.432^†^	3	1	0.882†
II	27	22		30	15	
III	3	6		5	4	
Stage						
I	9	5	0.420^†^	13	5	0.857†
II	13	17		12	8	
III	13	8		12	7	
IV	1	2		1	0	
Estrogen receptor						
Positive	21	17	0.807^†^	27	16	0.541†
Negative	15	15		11	4	
Progesterone receptor						
Positive	20	14	0.466^†^	25	16	0.366†
Negative	16	18		13	4	
Hormonal therapy						
No	17	13	0.631^†^	10	4	0.751†
Yes	19	19		28	16	
Surgery						
Mastectomy	22	18	0.711^†^	32	15	0.540†
Lumpectomy	14	13		5	5	
No surgery	0	1		1	0	
Chemotherapy						
None	3	2	0.702^†^	10	9	0.333†
Adjuvant/Neoadjuvant						
Anthracycline	20	20		23	8	
Anthracycline + Taxane	8	7		4	3	
CMF^§^	3	1		0	0	
Taxane	1	0		0	0	
Palliative						
Anthracycline	1	0		1	0	
Anthracycline + Taxane	0	2		0	0	
Trastuzumab therapy						
No	21	17	0.175^†^	N/A	N/A	N/A
Adjuvant	12	7				
Palliative	3	8				

*Student’s *t* test; ^†^Fisher’s exact test; ^§^CMF: classical CMF, cyclophosphamide, methotrexate, and fluorouracil

**Fig. 2:**
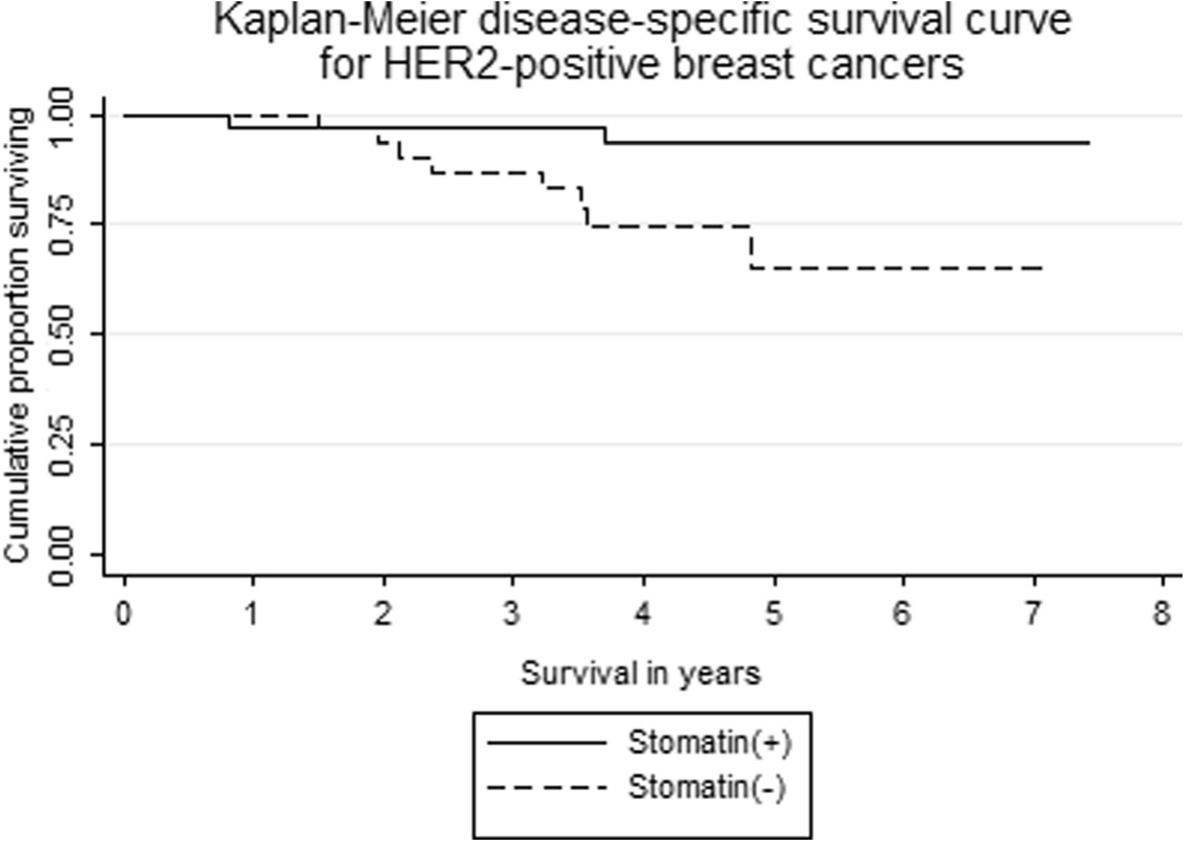
Kaplan-Meier method showing 5-year breast cancer-specific survival curves of HER2-positive infiltrating ductal carcinoma of breast comparing patients of positive and negative stomatin staining. Significant reduction of proportion surviving was noted in patients of stomatin (−) (*n* = 32), compared with patients of stomatin (+) (*n* = 36). *p* = 0.005

In the 68 women of stage I-IV HER2-positive infiltrating ductal carcinomas, mean follow-up time was 5.0 years. Kaplan-Meier plot (Fig. 2) showed that the 5-year breast cancer-specific survival rates were 93 % (95 % confidence interval = 76 to 98 %) for women of positive stomatin expression and 65 % for women of negative stomatin expression (95 % confidence interval = 38 to 83 %, *p =* 0.005). Namely, negative stomatin expression was significantly associated with a lower 5-year-survival rate. In comparison, there was no survival difference in patients with HER2-negative breast cancers. In women of HER2-negative cancers, the 5-year breast cancer-specific survival rates were 84 % (95 % confidence interval = 67 to 92 %) for women of positive stomatin expression and 94 % for women of negative stomatin expression (95 % confidence interval = 67 to 99 %, *p =* 0.193).

**Fig. 3:**
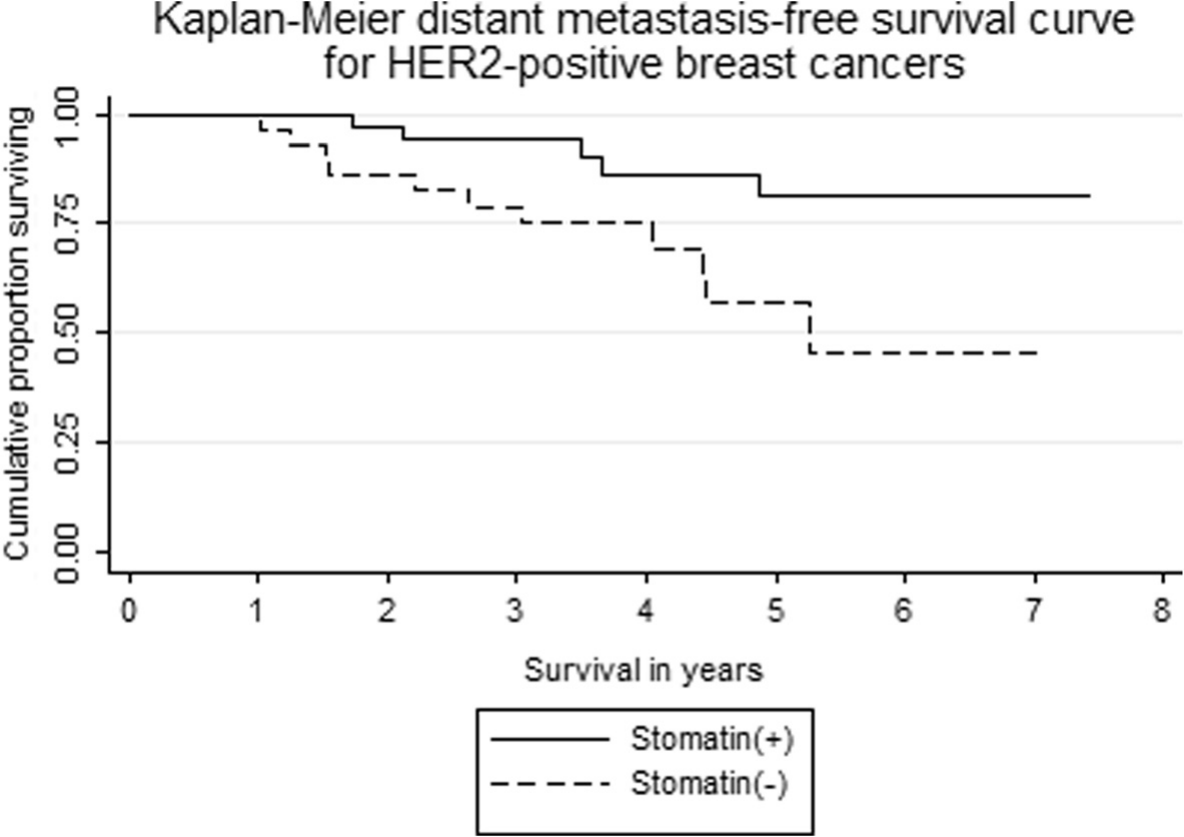
Kaplan-Meier method showing 5-year disease-free (distant metastases-free) survival curves of HER2-positive infiltrating ductal carcinoma of breast comparing patients of positive and negative stomatin staining. Significant reduction of proportion surviving was noted in patients of stomatin (−) (*n* = 30), compared with patients of stomatin (+) (*n* = 35). *p* = 0.016

In the follow-up of 65 women of stage I-III HER2-positive cancer, there were lung metastases in 5, bone metastases in 4, liver metastases in 3, lung & bone metastases in 1, lung & liver metastases in 2, distant lymph nodes metastases in 1, and local recurrence without a distant metastasis in two women. When the woman of local recurrence without a distant event was not regarded as failure of disease-free status, 5-year distant disease-free survival were 81 % (95 % confidence interval = 60 to 92 %) for women of positive stomatin expression and 57 % for women of negative stomatin expression (95 % confidence interval = 33 to 75 %, *p =* 0.016, Fig. 3). In comparison, there was no difference in patients with HER2-negative breast cancers. The 5-year distant distant disease-free survival rates were 64 % (95 % confidence interval = 46 to 77 %) for women of positive stomatin expression and 74 % for women of negative stomatin expression (95 % confidence interval = 46 to 88 %, *p =* 0.479) for HER2-negative patients.

For women of HER2-positive cancers, when either local recurrences or distant metastases were regarded as failure, the 5-year disease-free survival were 79 % (95 % confidence interval = 58 to 90 %) for women of positive stomatin expression and 59 % for women of negative stomatin expression (95 % confidence interval = 36 to 77 %, *p =* 0.037).

Although hormonal receptors expression, cancer stage, and adjuvant trastuzumab therapy were all known prognostic factors for HER2-positive breast cancer, multivariate analyses revealed stomatin was an independent factor for cancer metastases (*p* = 0.017, Table 2) for stage I-III HER2-positive breast cancers. Negative stomatin expression predicts distant metastases in a hazard ratio of 4.0 (95 % confidence interval from 1.3 to 12.5, Table 2).

**Table 2:** Multivariate Cox model for distant metastases-free survival in women of HER2-positive infiltrating ductal carcinoma, stage I-III (*n* = 65)

	Distant metastases	
Parameter	Risk ratio (95 % CI^a^)	*p*
Hormonal receptors^‡^		0.405
ER (+) or PR (+)	1.0 (referent)	
ER (−) and PR (−)	1.5 (0.6 to 4.3)	
Stage		0.016
I–II	1.0 (referent)	
III	3.5 (1.3 to 9.9)	
Adjuvant trastuzumab		0.147
Yes	1.0 (referent)	
No	3.1 (0.7 to 14.8)	
Stomatin expression		0.017
Positive	1.0 (referent)	
Negative	4.0 (1.3 to 12.5)	

^‡^ *ER* estrogen receptor, *PR* progesterone receptor

^a^ *CI* confidence interval

## Discussion

The present study provides evidence showing a correlation between stomatin protein expression and HER2-positive breast cancer prognosis. Compatible with a previous immunohistochemistry study, which showed that 31 % of the breast cancers were negative and 39 % weak for stomatin staining, the overall immunohistochemistry staining in our study showed negative staining in 47 % (32/68) of samples and positive staining in 53 % (36/68) of samples. Because there was no previous study to define the positivity according the expression level of stomatin, we defined the cutoff point of positive expression to be a staining index of 4 or more according to a preliminary tome-dependent ROC analysis. It was found that negative stomatin expression was associated with a decreased breast cancer-specific survival and disease-free survival using survival analyses. When distant metastases were defined as cancer recurrence for patients of stage I-III, stomatin was also an independent prognostic factor using multivariate analysis in this study.

Although stomatin-like protein 2, which is a member of the stomatin protein family, has been widely reported to be related to various kinds of cancers, there is only one report up to the present indicating an association between stomatin expression and carcinogenesis [19]. Arkhipova’s and colleagues recently reported that down-regulation of stomatin mRNA was correlated with positive lymph node status in 48 patients [19]. In our study, we initially proposed that lipid-raft localized stomatin might be able to modulate the activity of the HER2-positive breast cancer. The results that absence of stomatin expression might predict distant metastases in HER2-positive breast cancer are comparable to that of Arkhipova’s study for lung cancer [19].

One reason why previous researchers may have overlooked the relationship between stomatin and carcinogenesis may be because only a subgroup of breast cancers, namely the HER2-positive cancers, is affected by stomatin expression. In our study, when the 58 patients with HER2-negative breast cancer were analyzed, there was no survival difference between the stomatin-positive group and the stomatin-negative group. Although why stomatin down-regulation is associated with metastases in HER2-positive cancers remains uncertain, our results may suggest an interaction between HER2 receptor and stomatin in the lipid raft microdomains.

In Taiwan, 90 % of the invasive breast cancers are infiltrating ductal carcinoma and less than 4 % are infiltrating lobular carcinoma, with smaller percentages in other histology [31]. For reducing bias among different histologies, we chose women of the commonest histological type, infiltrating ductal carcinoma, as our study subjects. In our preliminary study for the histologies other than infiltrating ductal carcinoma, we can not make any conclusion in the influence of stomatin expression to cancer outcomes because the sample size is too small. Further large studies are needed to confirm the relationship between stomatin expression and disease outcomes in other histological types.

Gene expression patterns have distinguished several subtypes of breast carcinomas [32]. In a study reported by Prat el al., all molecular subtypes were identified in the 468 clinical HER2-positive tumors, including HER2-enriched (47 %), luminal B (28.2 %), basal-like (14.1 %), and luminal A (10.7 %) [33]. The molecular subtypes significantly affected survival outcomes [33, 34]. In our study, although stomatin was an independent factor in multivariate analysis, the molecular patterns of the tumors were unknown. Future studies exploring gene expression profiles are warranted.

By definition, disease-free survival usually includes local or regional recurrence in addition to distant metastases. However, the impact of local recurrences on survival remains uncertain. Local recurrence itself may be caused by failed local treatment but not by cancer progression. In this study, we defined disease-free survival as time to distant recurrence, excluding local or regional recurrence. Our study provided the valuable results that negative stomatin expression predicts distant metastases in a hazard ratio of 4.0 (95 % confidence interval from 1.3 to 12.5) in multivariate analyses.

In randomized controlled trials, patients receiving adjuvant trastuzumab showed a significant reduction in mortality and recurrence as compared to those of no adjuvant treatment [35]. The limitation of this study was that the adjuvant trastuzumab was not used for most women because of national insurance policy. Only women had positive lymph nodes were afforded adjuvant trastuzumab and this policy was effective after 2010. Because the patients who had ever received adjuvant trastuzumab were a minor group (28 %, 19 of 68, Table 1) and had a short follow-up time, the influence of trastuzumab treatment on stomatin expression could not be well explored. A clinical trial recruiting more patients is needed in the future. However, in the multivariate analyses, stomatin expression was an independent prognostic indicator from trastuzumab treatment, with a higher hazard ratio than that of adjuvant trastuzumab (hazard ratio 4.0 vs. 3.1). This result may suggest a different pathway of cancer progression in stomatin compared to that of HER2 receptor.

## Conclusions

Our findings suggest stomatin as one potential prognostic factor that predicts the progression in HER2-positive breast cancer. Further studies investigating the mechanism whereby stomatin affects HER2-positive breast cancer and how stomatin interacts with HER2 are needed.

## Abbreviations

AUC: Area under curve
CI: Confidence interval
CMF: Classical CMF chemotherapy, including cyclophosphamide, methotrexate, and fluorouracil
ER: Estrogen receptor
FISH: Fluorescence in situ hybridization
HER2: Human epidermal growth factor receptor 2
PR: Progesterone receptor
ROC: Receiver operating curve
